# Double Malignancy and Double Transplant—A Bumpy Road to Success

**DOI:** 10.3390/medicina59071209

**Published:** 2023-06-27

**Authors:** Michał Razik, Patrycja Rozwadowska, Anna Koclęga, Grzegorz Helbig

**Affiliations:** 1 Students’ Research Group, Department of Hematology and Bone Marrow Transplantation, Faculty of Medicine in Katowice, Medical University of Silesia, 40-055 Katowice, Poland; 2 Department of Hematology and Bone Marrow Transplantation, Faculty of Medicine in Katowice, Medical University of Silesia, 40-055 Katowice, Poland

**Keywords:** allogeneic stem cell transplantation, Ewing sarcoma, complications, immunosuppressive treatment, kidney transplantation, T-cell lymphoblastic lymphoma

## Abstract

The occurrence of secondary neoplasms in adult patients treated with chemotherapy in childhood is not uncommon. Prior chemotherapy is found to be an independent risk factor for the development of secondary malignancies, which are usually associated with a worse prognosis. The presented case is a 35-year-old female patient who was diagnosed with Ewing sarcoma in her late adolescence. The tumor was successfully treated with chemotherapy, but 3 years later she was diagnosed with T-cell lymphoblastic lymphoma. The patient received allogeneic hematopoietic stem cell transplantation (allo-HSCT) from human leukocyte antigen (HLA) matched related donor. The procedure was complicated by grade 2 acute graft-versus-host disease (GvHD) which resolved after implementation of immunosuppressive treatment. However, a year later, the patient developed extensive chronic GvHD (cGvHD) and required reintroduction of immunosuppressants. Prolonged immunosuppressive treatment with tacrolimus led to irreversible kidney failure. After a 2-year period of regular peritoneal dialysis, she was found to be eligible for a kidney transplant from a deceased donor. Now, 15 years after stem cell transplantation and 8 years after kidney transplantation, the patient remains in good condition overall, presenting with symptoms of limited cGvHD. The case described here presents a unique clinical scenario of a female patient who was successfully treated for her double malignancy. Moreover, she underwent effective double transplantations and was eventually found to be cured despite accompanying complications.

## 1. Introduction

Along with improving effectiveness of available methods of treatment and prolonged survival rates, one should be aware of the possibility of developing neoplasms secondary to the previous oncological treatment [1]. They may appear shortly or many years after the end of treatment [2]. Data from the Surveillance, Epidemiology, and End Results (SEER) registry indicate that the cumulative incidence of developing secondary hematological malignancy at 5 and 10 years after primary diagnosis is 0.2% and 0.4%, respectively [3]. The highest numbers are observed in childhood cancer survivors (observed/expected ratio, 3.47; 95% CI, 2.86–4.18). Specifically, there was an increased incidence of acute myeloid leukemia (excess risk, 1.46 per 10,000), acute lymphoblastic leukemia (excess risk, 0.3 per 10,000), and non-Hodgkin lymphoma (excess risk 0.51 per 10,000). A population-based study revealed a higher incidence of secondary acute leukemias in patients treated for sarcoma, particularly in those with Ewing sarcoma and osteosarcoma [4]. Some agents, e.g., taxanes or topoisomerase inhibitors, when compared with other anticancer agents, were found to increase the risk of secondary malignancies [5]. Moreover, a relationship between administered doses of radio- or chemotherapy and morbidity has been demonstrated [6]. When compared with their primary counterparts, therapy-related neoplasms are associated with inferior 5-year survival [7]. *TP53* and *PPM1D* mutations have been demonstrated to have a statistically significant relationship with exposure to both chemo- and radiotherapy [8]. Well-characterized in other hematological malignancies, mutant-type *TP53* plays a role in the development of lymphoid neoplasms and is associated with poor prognosis [9]. Although not yet settled, *PPM1D* mutations have been found to contribute to clonal hematopoiesis, and chemoresistance to specific agents [10]. Hematologic malignancies secondary to the previous oncological treatment can be characterized by prognostically unfavorable genetics, thus poor prognosis. Therefore, further treatment with allogeneic hematopoietic stem cell transplantation (allo-HSCT) may be required whenever possible.

The case described here presents a unique clinical scenario of a female patient who was successfully treated for her double malignancy. Moreover, she underwent effective double transplantations and eventually was found to be cured despite accompanying complications.

## 2. Case Presentation

The patient reported here is a 35-year-old female, with a previous history of Ewing sarcoma of the right tibia diagnosed at the age of 17. The disease was treated with chemotherapy consisting of six courses of vincristine, doxorubicin, ifosfamide, and etoposide (VIDE), followed by seven courses of vincristine, dactinomycin, and ifosfamide (VAI), and completed with a radical knee joint resection with prosthetic reconstruction. Three years later, in 2007, the patient was diagnosed with T-cell lymphoblastic lymphoma, with a tumor mass located mainly in mediastinum. No blast cells in peripheral blood and bone marrow were observed. Induction and consolidation regimens were administered as per Polish Adult Leukemia Group (PALG ALL 4-2002) protocol [11]. Central nervous system (CNS) prophylaxis consisted of six lumbar punctures with intrathecal administration of methotrexate. Cerebrospinal fluid was lymphoma-free. Complete remission (CR) was obtained and confirmed in follow-up computed tomography (CT) scans. Then, the patient was scheduled for allogeneic hematopoietic stem cell transplantation (allo-HSCT). In May 2008, bone marrow stem cells from a related donor—a 26-year-old, human leukocyte antigen (HLA) fully matched brother—were infused. Conditioning regimen consisted of cyclophosphamide and total body irradiation (TBI). Graft-versus-host disease (GvHD) prophylaxis included methotrexate and cyclosporine A (CsA).

Post-transplant aplasia was complicated by mild oral mucositis. On day +16 the patient demonstrated symptoms of grade 2 acute GvHD (aGvHD) and received methylprednisolone (MP) at 0.5 mg/kg daily. Bone marrow aspiration on day +29 showed features of marrow regeneration and full donor chimerism. MP was stopped on day +64, while CsA was continued. As skin and mucosal symptoms subsided, the dose of CsA was gradually reduced and discontinued 6 months after transplantation (December 2008). A CT scan of the prosthesis area was performed and no pathological contrast enhancement suggesting the recurrence of Ewing sarcoma was demonstrated. At a follow-up visit in May 2009, the patient reported general weakness, pain and stiffness in the lower limbs, fragility of the nails, erythema and itching of the skin. On physical examination, swelling of the ankles and lower extremities and diffuse skin hyperpigmentation were noted. The patient was diagnosed with cutaneous and articular manifestations of chronic GvHD (cGvHD), and CsA with MP were re-introduced. In October 2009, despite the immunosuppressive treatment, the progression of skin lesions in the form of scleroderma was observed. Mycophenolate mofetil (MMF) was added to CsA and MP. In July 2010, due to a limited effectiveness of the previous immunosuppressive treatment, CsA was switched to tacrolimus (Tac). As a result of the triple immunosuppressive therapy a slight regression of scleroderma lesions was demonstrated. Blood Tac concentrations were maintained at the therapeutic range.

The patient was admitted to the emergency department in May 2011, with a history of diarrhea, nausea, and vomiting after meals. Laboratory testing revealed uremia (94 mg/dL), increased creatinine level (3.77 mg/dL) and elevated C-reactive protein (CRP; 30.2 mg/L). An infectious etiology (*C. difficile*, *Salmonella*, *Shigella*, *Yersinia*, *E. coli*) of the presented symptoms was ruled out. Due to elevated Tac trough level (26 ng/mL), the drug was discontinued while MP and MMF were sustained. As no improvement in renal parameters was observed, the patient was qualified for peritoneal dialysis. After a 2-year period of regular peritoneal dialysis, a deceased donor kidney transplantation (KTx) was performed in March 2015 with basiliximab as prophylaxis. The procedure was free of complications. Laboratory testing on day +1 showed a significant decrease in creatinine (from 4.8 to 0.78 mg/dL) and eGFR normalization (from 11 to 89 mL/min/1.73^2^). At the last follow-up visit, the patient presents limited cutaneous symptoms of cGvHD, but her overall condition is good. Biochemical tests show no abnormalities. Positron emission tomography-computed tomography (PET-CT) scan detects no signs of recurrence of Ewing sarcoma as well as lymphoma.

## 3. Discussion

Due to T-cell lymphoblastic lymphoma, the patient underwent an allo-HSCT procedure, which can be associated with an increased risk of renal dysfunction. This is mainly due to the use of renal toxic immunosuppressive agents and infections [12]. Other independent risk factors include thrombotic and endothelial complications of the peri-transplant period, particularly hepatic veno-occlusive disease (VOD) [13,14]. These can be a great challenge in clinical practice and require a balance between efficacy and toxicity, when choosing the appropriate management [15]. Therefore, it seems reasonable to ensure regular check-ups for both current and previous diseases in order to effectively control them, but at the same time avoid burdensome side effects [16]. Due to the significant nephrotoxicity of the drugs in common use, especially calcineurin inhibitors, irreversibly deteriorating kidney function may require proper management including hemodialysis followed by kidney transplantation [17]. The presented case gives a clear example of rapidly developed renal dysfunction following the administration of Tac, as indicated by its exceeded blood level, far beyond the therapeutic index (TI). With calcineurin inhibitors—CsA and Tac—remaining the most widely used drugs in GvHD prophylaxis, it is necessary to implement effective solutions to avoid their nephrotoxicity, and in the event of its occurrence—adequate management. There have been attempts to replace traditional calcineurin inhibitors with alternative drugs. One of them is cyclophosphamide (CTX), originally used as an anti-cancer medication. It was recently shown that post-transplant CTX decreased the incidence of severe GvHD (both acute and chronic), and it translated into better relapse-free and GvHD-free survivals when compared with standard prophylaxis [18].

Another potential substitute for calcineurin inhibitors is sirolimus. Clinical studies suggest that it may be an effective and well-tolerated immunosuppressive therapy with a better safety profile, especially in terms of renal toxicity [19]. The introduction of new therapeutic options could contribute to better personalized immunosuppressive treatment. The main goal is to reduce the risk of side effects and improve long-term outcomes. As for today, due to the lack of sufficient data regarding the wider use of novel agents, it seems obvious to minimize the dose of calcineurin inhibitors and keep their level within the TI [20].

Effective treatment of hypertension remains crucial to decrease both the risk of cardiovascular disease and the progression of renal failure. Bearing in mind the pathomechanism, i.e., vasoconstriction of the afferent arteriole and subsequent prerenal injury, the use of dihydropyridine Ca-blockers seems to be a promising first-line treatment for hypertension following allo-HSCT. There is evidence of their impact on the significant reduction in blood pressure and concomitant improvement in creatinine clearance [21]. Angiotensin-converting enzyme inhibitors (ACEIs) and angiotensin II receptor blockers (ARBs) should also be taken into consideration [22]. Due to their mechanism of action, they may contribute to a significant reduction of proteinuria [23]. Being a common occurrence after transplantation, its presence is associated with further renal damage and disease progression. However, it is essential to emphasize that the decision on the use of ACEIs or ARBs after allo-HSCT should be individualized and discussed with a multidisciplinary team. Additional therapeutic options include dietary and lifestyle modifications [24].

As conventionally used measures failed, the presented patient required long-term hemodialysis. No consensus has yet been established on the right timing of dialysis initiation. Data obtained from studies with pediatric population indicated the benefits of early implementation of balanced fluid therapy and hemodialysis [25,26]. However, as another study showed, hemodialysis in patients after allo-HSCT can be associated with a high mortality rate [17]. In studies involving children who had received allo-HSCT, the survival rate was higher in patients with convective therapy (continuous venovenous hemofiltration or hemodiafiltration) than among those with diffusion therapy (dialysis) [27].

A retrospective multicenter study has been performed on solid organ transplantation following allo-HSCT involving 13 patients receiving KTx [28]. In this group, the main complication was organ rejection affecting four patients (31%), of which two (15%) developed another terminal renal failure requiring retransplantation. Nevertheless, the overall survival was 100% at 5 years. Thus, the study indicates that KTx may be a rational therapeutic approach in selected young patients. Overall, organ survival rates were comparable to patients undergoing transplantation for other reasons. At the same time, taking into consideration the total number of allo-HSCT performed in the participating centers and the general frequency of organ failure as its complication, it is worth noting that this procedure is yet rarely performed. The presented patient is another example of successful KTx as a procedure to reverse the toxic effects of immunosuppressive drugs. Nevertheless, due to its significant invasiveness, careful monitoring and early intervention are preferably required to prevent this complication.

The presented case highlights another problem faced by contemporary oncology. Early adolescent and young adult cancer survivors require long-term screening for chronic health conditions. These are known to develop at a higher risk than in the general population [29]. It seems that the transitional period between childhood and adulthood remains an underestimated gap in many cancer care systems, thus affecting the quality of life of patients. The role of onco-nephrologists seems to be invaluable in managing patients before and after allo-HSCT [30]. This involves identifying patients at a higher risk of kidney injury, where even a slight change in serum creatinine (Cr) levels may be present. Deteriorating renal function requires proper interventions, such as adjusting drug dosages to prevent nephrotoxicity, addressing fluid and electrolyte imbalances, and early initiation of renal replacement therapy. Additionally, it is crucial to routinely evaluate albuminuria and/or proteinuria [31]. Attempts have already been made to work out clear evidence-based recommendations for the follow-up care of such patients [32]. As suggested for the pediatric population after HSCT, frequent monitoring should be performed for the first 12 months followed by less frequent follow-up, thereafter, depending on the individual’s kidney function [33]. Screening for early diagnosis and treatment of kidney injury may be performed using traditional markers (e.g., Cr). It can be combined with biomarkers of glomerular function (e.g., serum cystatin C) and tubular injury (e.g., urinary neutrophil gelatinase-associated lipocalin [NGAL]) for higher sensitivity and specificity in the early post-transplant period [34,35]. So far, in the absence of developed standards of care for early adolescent and young adult cancer survivors, it has become necessary to systematize follow-up visits with periodic assessment of possible complications. This will allow for the prevention, early detection, and effective treatment, thereby improving the length and quality of life of patients. At the same time, efforts should be made to find new treatment methods that maintain the highest possible efficacy, but with the lowest possible side effect profile.

## 4. Conclusions

The occurrence of secondary neoplasms in adult patients treated with chemotherapy in childhood is not uncommon. Undoubtedly, chemotherapy is a significant risk factor for secondary malignancies. These have poorer survival rates compared to the primary. Early diagnosis and constant follow-up of the patient through regular visits and examinations can detect cancer at an early stage and take appropriate treatment. Also, early adolescent and young adult cancer survivors require long-term screening for distant complications such as chronic health conditions, including kidney injury. The transitional period between childhood and adulthood in cancer care systems needs better attention to reduce healthcare costs, and what is even more important, improve the quality of life of patients. The presented case shows how important it is to provide patients with continuous multidisciplinary care by, for example, creating oncology outpatient clinics for adults with childhood oncological diseases. It also points to the need for further research into alternative methods of treatment to reduce its potential side effects.

## Data Availability

The data presented in this study are available on request from the corresponding author.
